# Supplementary material for: Bimetallic Aluminum‐ and Niobium‐Doped MCM‐41 for Efficient Conversion of Biomass‐Derived 2‐Methyltetrahydrofuran to Pentadienes
Source: Angew Chem Weinheim Bergstr Ger. 2022 Nov 17;134(51):e202212164. doi: 10.1002/ange.202212164 (PMC10946597; doi:10.1002/ange.202212164)
Supplement: Supplementary file 1 — Supporting Information [file ANGE-134-0-s001.pdf]

Angewandte Chemie
Eine Zeitschrift der Gesellschaft Deutscher Chemiker

Supporting Information

Bimetallic Aluminum- and Niobium-Doped MCM-41 for Efficient Conversion of Biomass-Derived 2-Methyltetrahydrofuran to Pentadienes

M. Fan, S. Xu, B. An, A. M. Sheveleva, A. Betts, J. Hurd, Z. Zhu, M. He, D. Iuga, L. Lin, X. Kang, C. M. A. Parlett, F. Tuna, E. J. L. McInnes, L. L. Keenan, D. Lee, M. P. Attfield*, S. Yang**

Contents

Experimental Section 4
Catalyst characterization. 4
Catalyst preparation. 4
Catalytic testing. 5
Electron Paramagnetic Resonance Spectroscopy. 6
Solid-state Nuclear Magnetic Resonance Spectroscopy. 6
In situ combined EDE/DRIFTS measurement for AlNb-MCM-41(35/1/0.9). 6

Figure S1. Picture of the set-up of I20-EDE beamline at Diamond Light Source. 8

Figure S2. Comparison of PXRD patterns of AlNb-MCM-41 and Al-MCM-41 (λ = 1.5406 Å), confirming the absence of Nb_2O_5. 8

Figure S3. N_2 adsorption isotherms of catalysts. (a) Al-MCM-41(35/1), (b) AlNb-MCM-41(35/1/0.3), (c) AlNb-MCM-41(35/1/0.7), and AlNb-MCM-41(35/1/0.9) at 77 K. 9

Figure S4. TEM images of catalysts. (a) Al-MCM-41(35/1), (b) AlNb-MCM-41(35/1/0.3), and (c) AlNb-MCM-41(35/1/0.7). 9

Figure S5. The O 1s deconvoluted spectrum of AlNb-MCM-41(35/1/0.9). 10

Figure S6. ^{27}Al (left) and ^{1}H (right) MAS NMR spectra of Al-MCM-41 (35/1), Al-MCM-41 (35/1) with 2-MTHF, AlNb-MCM-41(35/1/0.9) and AlNb-MCM-41(35/1/0.9) with 2-MTHF. 11

Figure S7. (a) Wide-field X-band EPR spectra of the activated molecular sieves after γ-irradiation detected at 40 K; (b) narrow-field X-band EPR spectra of the activated molecular sieves after γ-irradiation detected at 40 K. 12

FigureS8. Characterisation of the acidity of catalysts by NH_3-TPD. 12

Figure S9. Characterization of the acidity of catalysts by d_3-acetonitrile-IR. 13

Figure S10. Acetone DRIFTS spectra. 14

Figure S11. Analysis of the product for the conversion of 2-MTHF for 8 h on AlNb-MCM-41(35/1/0.9). (a), GC trace of the gaseous product detected by TCD, column: PLOT-U. (b), GC(FID) trace of the liquid product diluted by acetone. 1,4-Dioxane was used as internal standard; FID, column: HP-5. It could be confirmed that the conversion for 2-MTHF >99% with only trace amounts of 2-MTHF detected. (c) GC-MS analysis of the gaseous product and (d) the corresponding mass spectrum of 1,3-pentadiene product. 15

Figure S12. Simulated IR spectrum of trans-1,3-pentadiene. 16

Figure S13. Fitting of EXAFS data at the Nb K-edge absorption 17

Figure S14. On-line mass spectra of 2-MTHF and pentadienes during conversion of 2-MTHF on AlNb-MCM-41(35/1/0.9), for *in situ* DRIFTS and Nb K-edge XAS experiments. 18

Figure S15. GC-MS analysis of the liquid products generated in a batch reactor over NbAl-MCM-41(35/1/0.9) at different time. (a) 0.5 h, (b) 1h, (c) 1.5 h, and (d) 2h. (e) Yields of different intermediates at different time. Reaction condition: 2-MTHF, 2 ml; catalyst, 0.1 g; reaction temperature, 220 ^{o}C. 19

Table S1. Specific surface areas of all zeolites used in this study (data determined from N_2 sorption isotherms at 77 K) 20

Table S2. Summary of acidity of all MCM-41 materials used in this work 20

Table S3. Spin-Hamiltonian parameters of radiation induced Nb(IV) and electron hole Al-O$^{\bullet}$-Si defects, from simulation of CW X-band EPR spectra in FigureS7: lw is the homogeneous Lorentzian linewidth, g and A are the principle values of the g and hyperfine interaction tensors. 21

Table S4. Comparison of the catalytic performance between AlNb-MCM-41 and reported catalysts for the conversion of 2-MTHF to pentadienes. 21

Table S5. 1st Shell fitting of EXAFS data on the Nb sites for as-prepared and 2-MTHF-adsorbed AlNb-MCM-41. 22

References: 23

Experimental Section

Catalyst characterisation.

Small-angle X-ray scattering patterns were collecting on a Rigaku FR-X dual wavelength rotating anode diffractometer with an AFC-11 4-circle goniometer, VariMAX™ microfocus optics and a Hypix 6000-HE area detector, using CuKα radiation (λ = 1.54184Å), beam divergence of 1.5mR and detector distance of 150mm. Data were collected at detector theta angles of 5°, 0°, -22.8° and -45.7° with 300° 300s phi scans at each detector position. Data were collected and integrated using the powder extraction tool in Crysalispro v171.41. Powder X-ray diffraction (PXRD) patterns were recorded on a Philips X'pert X-ray diffractometer (40 kV and 30 mA) using Cu Kα radiation (λ=1.5406 Å). N_2 adsorption was carried out at 77 K on a Micromeritics 3Flex instrument after activating the samples for 12 h under dynamic vacuum at 453 K. The crystal morphology and size were measured by scanning electron microscopy (SEM) on a Quanta FEG 650 microscope. The ratios of Si/Al/Nb in the sample were quantified by EDX on multiple regions over a sample on a Bruker XTrace instrument. The morphologies of the samples were characterized by the transmission electron microscope (JEOL-1011).

The acidity concentration was measured by temperature-programmed desorption of ammonia (NH_3-TPD) with a Quantachrome Autosorb-1 equipped with a thermal conductivity detector (TCD). Typically, 100 mg of sample was pre-treated in helium stream (30 mL min^{-1}) at 600 °C for 2 h. The adsorption of NH_3 was carried out at 50 °C for 1 h. The catalyst was flushed with helium at 100 °C for 2 h to remove physisorbed NH_3 from the catalyst surface. The TPD profile was recorded at a heating rate of 10 °C min^{-1} from 100 to 600 °C. The Brønsted and Lewis acid sites of the samples were investigated by *in situ* DRIFTS of adsorbed acetonitrile-d_3. Wafers with a weight of 30 mg were active for 2 h under N_2 flow at 550 °C. Then acetonitrile-d_3 was admitted by bubbler system, and after equilibration, the samples were purged by N_2 flow for 0.5 h upon heating at 35, 200, and 250°C. The oxygen affinity of catalysts was verified by acetone adsorption-desorption DRIFTS experiment. Wafers with a weight of 30 mg were active for 2 h under N_2 flow at 550 °C. Then acetonitrile-d_3 was admitted by bubbler system, and after equilibration, the samples were purged by N_2 flow for 0.5 h upon heating at 35, 120, and 150 °C. X-ray photoelectron spectroscopy (XPS) was performed on an Axis Ultra Hybrid spectrometer (Kratos Analytical, Manchester, United Kingdom) using monochromated Al Kα radiation (1486.6 eV, 10 mA emission at 150 W, spot size 300 x 700 μm) with a base vacuum pressure of $\sim 5 \times 10^{-9}$ mbar. Charge neutralisation was achieved using a filament. Binding energy scale calibration was performed using C-C in the C 1s photoelectron peak at 285 eV. Analysis and curve fitting was performed using Voigt-approximation peaks using CasaXPS.

Catalyst preparation.

AlNb-MCM-41, Al-MCM-41, Nb-MCM-41 and MCM-41 materials were synthesized using a modified method based upon a previous report[1], and denoted as AlNb-MCM-41 (Si/Al/Nb mole ratio), Al-MCM-41 (Si/Al mole ratio), Nb-MCM-41 (Si/Nb mole ratio) and MCM-41(Si), respectively. In a typical synthesis, hexadecyltrimethyl-ammonium bromide (CTABr, 99+%, Sigma Aldrich) was used as the template reagent and

firstly dissolved in a solution of deionized water, and tetrapropylammonium hydroxide (TPAOH, 1.0 M in H_2O, Sigma Aldrich) which is an agent for directing Al^{3+} ion in the tetrahedral coordination environment. Then the mixture was stirred for 2 h, which was followed closely by the addition of aluminium isopropoxide (99.99+%, Sigma Aldrich) with stirring for 2 h at room temperature. Niobium ethoxide (99.95%, Sigma Aldrich) was added and the mixture was stirred for another 2 h. Then tetraethyl orthosilicate (98%, Sigma Aldrich) was added dropwise and the mixture stirred for another 2 h, resulting in a gel with a chemical composition of 35Si: xAl: yNb: 4.2CTABr: 5.6TPAOH: 595H_2O (x and y were determined by the target Si/Al and Si/Nb mole ratios, respectively). The gel was transferred into a 50-mL Teflon-lined stainless-steel autoclave, which was sealed and heated at 100 °C for 3 days. The solid products were centrifuged, washed with deionised water, dried overnight at 80°C, and finally calcined at 550°C under an air flow for 14 h. Al-MCM-41 samples were synthesised by the same procedure but without addition of niobium ethoxide. Nb-MCM-41 samples were synthesised by the same procedure but without addition of aluminium isopropoxide. $NbOPO_4$ was synthesized by a hydrothermal method according to a reported method[2]. Nb_2O_5 was purchased from Sigma Aldrich.

Catalytic testing.

Catalytic reactions were carried out in a stainless steel continuous-flow reactor (12.7 mm i.d.). 0.5 Gram of the catalyst was pressed, crushed, and sorted into grains in range of 40–60 meshes, which were then activated at 550 °C for 3 h under a N_2 flow before the reaction. 2-MTHF was injected into the N_2 flow by syringe pump (Cole-Parmer) and passed through the reactor at the target temperature. The output liquid products were collected and analysed by GC (Agilent 7890B, equipped with an HP-5 column 30 m × 0.32 mm × 0.25 μm) and GC-MS (Agilent 6890A–Agilent 5973N, equipped with an HP-5MS column 30 m × 0.25 mm × 0.25 μm). The output gas products were collected and analysed by GC (Agilent Micro GC 490 equipped with a PoraPLOT U column, length 10 m) and GC–MS (Agilent 7890A-Agilent 5975C, equipped with an HP-PLOT/Q column 30 m × 0.53 mm × 40 μm). The conversion of 2-MTHF is calculated by:

$$\text{2-MTHF conversion} = (\text{2-MTHF}_{in} - \text{2-MTHF}_{out}) / \text{2-MTHF}_{in} \times 100\%$$

The selectivity is calculated by:

$$\text{Selectivity}_i \text{ (carbon basis)} = \text{Total carbon present in the product}_i / \text{total carbon from the converted reactant.}$$

Batch reactions were conducted in a Teflon-lined stainless-steel autoclave (10 mL) equipped with a temperature-controlled heating block and magnetic stirring. In a typical procedure, 100 mg of NbAl-MCM-41(35/1/0.9) and 2 mL of 2-MTHF were charged into the autoclave, which was then sealed and heated to a target temperature under stirring at 600 rpm for a given period of time. After the reaction, the system was quenched by an ice bath and the liquid phase was collected for analysis. The liquid products were collected and analysed by GC (Agilent 7890B, equipped with an HP-5 column 30 m × 0.32 mm × 0.25 μm) and GC-MS (Agilent 6890A–Agilent 5973N, equipped with an HP-5MS column 30 m × 0.25 mm × 0.25 μm).

The yield is calculated by:

$$\text{Yield}_i = \text{mole of the product}_i / \text{mole of 2-MTHF(initial)} \times 100\%.$$

Electron Paramagnetic Resonance Spectroscopy.

For the EPR spectroscopy, the sample was placed in a 4.0 mm o.d. quartz tube and connected to a vacuum line. Prior to irradiation, catalyst materials were activated by pumping at 10^{-5} Torr for 12 h at 150 ˚C and then flame sealed under vacuum. The samples were exposed to γ-irradiation from a ^{60}Co source at 77 K to a total dose of 4.42 MRad at a dose rate of 0.34 MRad h^{-1}. CW EPR measurements were carried out at X-band (~9.4 GHz) using a commercial spectrometer Bruker EMX equipped with Oxford Instruments temperature control system at 40 K. EPR spectra were detected with modulation amplitudes of 0.1 and 1 mT, and microwave powers varied in the range ~0.7-70 mW. For the data presented here, 7 mW was chosen (if it was not discussed additionally in the text) to provide optimum signal intensity without saturation of spectral lines. Theoretical modelling of all EPR data was performed using EasySpin toolbox (Version 5.2.33) for Matlab[3].

Solid-state Nuclear Magnetic Resonance Spectroscopy.

NMR spectra were acquired at the UK High-Field Solid-State NMR Facility on a 20.0 T (850 MHz ^{1}H Larmor frequency) Bruker AVANCE NEO spectrometer equipped with a 1.3 mm HXY MAS probe in double-resonance ^{1}H/^{27}Al mode, and a 4 mm HXY MAS probe in double resonance ^{1}H/^{93}Nb mode. ^{1}H and ^{27}Al NMR spectra were acquired at ambient temperature using a MAS frequency of 60 kHz, ^{1}H (π/2)-pulses of 2.5 μs, and ^{27}Al hard pulses of 1 μs. ^{1}H NMR spectra were recorded after a Hahn-echo ($\tau - \pi - \tau$), with a total echo time (2τ) of two rotor periods. 32 transients were co-added with inter-scan repetition delays of 1 s. ^{27}Al NMR spectra were recorded with 8192 co-added transients with inter-scan repetition delays of 0.2 s. ^{93}Nb NMR spectra were acquired under static conditions using a solid echo ($(\pi/2)_x - \tau - (\pi/2)_y - \tau$) with (π/2)-pulses of 1.5 μs and total echo time (2τ) of 40 μs. 32880 and 140512 transients were co-added with inter-scan repetition delays of 0.5 s, for the pristine and 2-MTHF-dosed AlNb-MCM-41(35/1/0.9), respectively. All samples were activated for 48 hours prior to packing into 4 mm o.d. zirconia NMR rotors. Following this, for the adsorbed samples, 2-MTHF was added and drying was repeated to remove any excess within the rotor in a vacuum oven at 60°C for at least an hour. Spectral simulations were performed in the solid lineshape analysis (SOLA) module v2.2.4 in Bruker TopSpin v4.0.9 and using Dmfit[4] for a Gaussian Isotropic Model to represent the distribution in isotropic chemical shift and quadrupolar parameters. The ^{1}H NMR chemical shifts were referenced to neat TMS externally, the ^{27}Al chemical shifts were referenced externally to 1.1 mol/kg $Al(NO_3)_3$ in D_2O, and the ^{93}Nb chemical shifts were referenced externally to a saturated solution of $NbCl_5$ in acetonitrile.

***In situ* combined EDE/DRIFTS measurement for AlNb-MCM-41(35/1/0.9).**

A combined energy dispersive EXAFS (EDE) and diffuse reflectance infrared Fourier transform spectroscopy (DRIFTS) measurements have been carried out on the I20-EDE beamline at Diamond Light Source, Harwell Innovation Campus, Oxfordshire, UK.[5] The storage ring energy was 3 GeV and was operated at 300 mA in top-up mode (with an injection period of 20 seconds every 10 mins. I20-EDE is an energy-dispersive EXAFS beamline that was optically optimised at the Nb absorption edge (18986 eV), in transmission mode. The synchrotron radiation from an 8 pole wiggler insertion device (18.5 mm gap between magnets (1.2 T)) is conditioned with an initial aperture of size 0.77 mm x 0.84 mm (h x v) and beam defining primary slits of 0.5

mrad horizontally and 0.1 mrad vertically. In horizontal the beam is focused using a curved Si(311) polychromator (radius of curvature = 12.0 m) in Bragg geometry.[6] This curvature leads to polychromatic beam with the energy range at the sample measured was 18807 to 19708 eV. The beam is vertically focused by a curved mirror ME1 at 36m from the beam source (radius of curvature = 8.6 km) with a Pt coating and 3.1 mrad pitch angle. The unwanted low energies were filtered out using a 2.8 mm thick pyrocarbon filter and two Be windows of 300 um thickness along the beamline. This gave a beam size of 90 x 200 um (FWHM h x v) at the focal spot on the sample position. To remove the background a reference sample of MCM-41Al without Nb was measured before each XAS measurement. The detector used was the FReLoN CCD (14 μm/pixel),[7] with a typical measurement of 7143 accumulations at 600 us each for a total of 10 seconds per spectrum. The detector's pixel to energy calibration was calculated by measuring a 25 μm thick Nb foil and comparing to the same foil measured on the monochromatic XAS beamline, B18, at Diamond Light Source. and rechecked daily during the experiment. The overall result is that the whole X-ray absorption spectrum to be collected simultaneously. Additionally, the size of the focussed beam at the sample position is very stable (+/-1 um horizontal and +/-10 um vertical beam position) as there is no requirement for the optical elements to be moved during the data collection. Simultaneous DRIFTS was facilitated through integration of a Bruker Invenio R spectrometer on the beamline, mounted on an in house designed trolley and stage to deliver the IR beam to the sample (FigureS1). The IR beam was focused from an external port to a Harrick Praying Mantis DRIFTS accessory mounted on an external optics board and aligned to an MCT detector. The set-up utilised a bespoke high temperature IR cell, fitted with 2 mm ZnSe IR windows, and one 0.5 mm glass carbon window and one 1 mm diamond window for transmission XAS. The solid sample was contained within a heated ceramic crucible, with glass flow configuration through the catalyst bed.

Measurements were employed as the following steps: 1). *In situ* activation of the sample at 550 °C in N_2 (30 mL min^{-1}) flow for 120 min. (ramp rate 5 °C min^{-1}). 2) Cooled down the sample in N_2 (30 mL min^{-1}) flow to room temperature. Then flowed 2-MTHF saturated in N_2 (10 mL min^{-1}) into the cell through a bubbler system. 3). Temperature programmed reaction of the sample in a constant flow of 2-MTHF saturated in N_2 (10 mL min^{-1}) from 30-300°C (1 °C min^{-1}), and then maintained for 120 min. Time-resolved Nb K-edge (18986 eV) XAS (6s per scan) measurements were collected in the range of 30-275 °C (ramp rate 1 °C min^{-1}). 4) Pure N_2 (30 mL min^{-1}) was flowed into the cell for 60 min to remove all adsorbed species on Nb(V) sites. The complementary coupling of surface infrared (DRIFTS), average spectra collected every ~60 seconds, probed the chemical speciation of adsorbates, whilst MS enabled real-time activity and selectivity to be monitored.

Processing the time resolved EDE data was made using the DAWN software package[8] in order to crop, calibrate and normalise the XAS spectra. The processing of the Extended X-ray Absorption Fine Structure (EXAFS) data was performed using IFEFFIT with the Horae package (Athena and Artemis)[9]. Athena was used to calibrate, align and normalise the spectra with respect to the Nb foil.

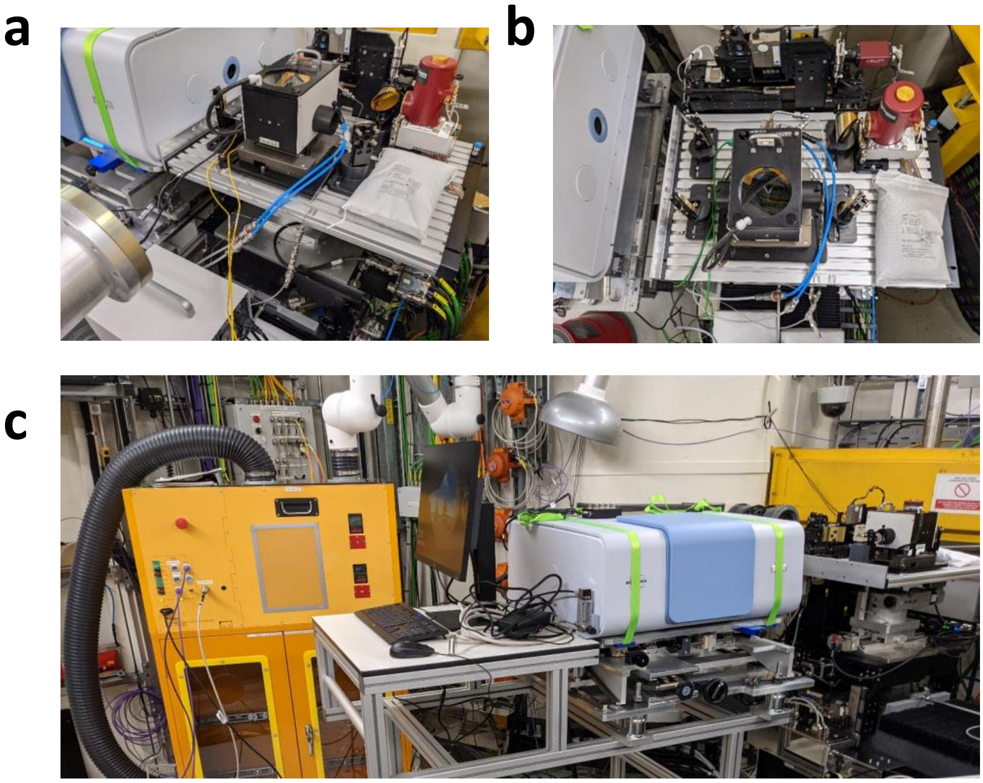

Figure S1. Picture of the set-up of I20-EDE beamline at Diamond Light Source.

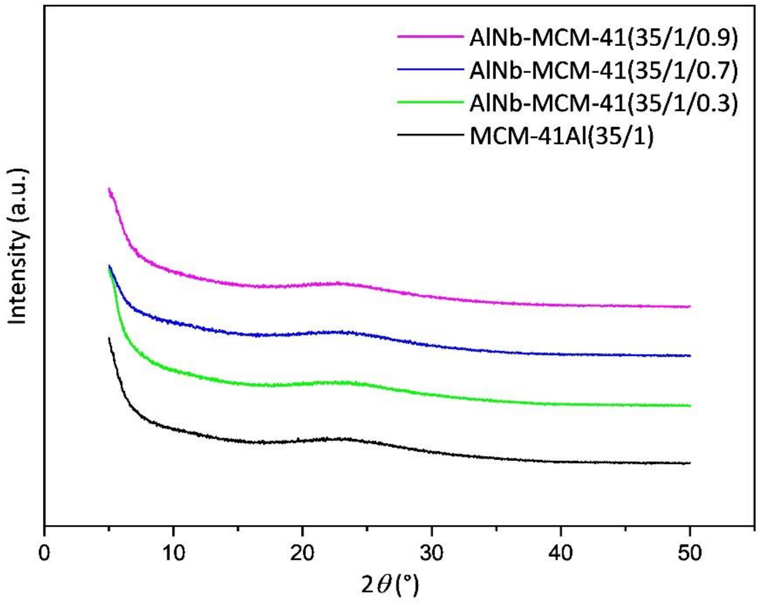

Figure S2. Comparison of PXRD patterns of AlNb-MCM-41 and Al-MCM-41 (λ = 1.5406 Å), confirming the absence of Nb_2O_5.

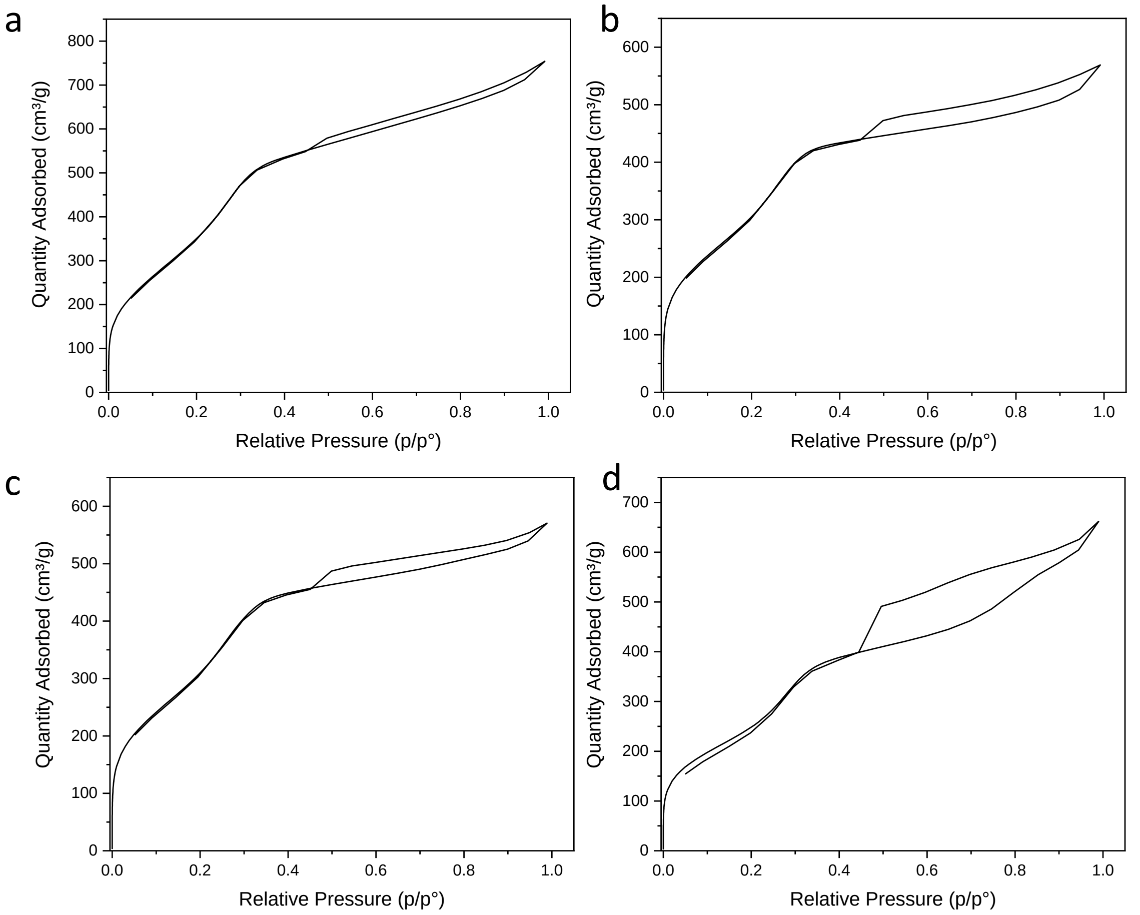

Figure S3. N_2 adsorption isotherms of catalysts. (a) Al-MCM-41(35/1), (b) AlNb-MCM-41(35/1/0.3), (c) AlNb-MCM-41(35/1/0.7), and AlNb-MCM-41(35/1/0.9) at 77 K.

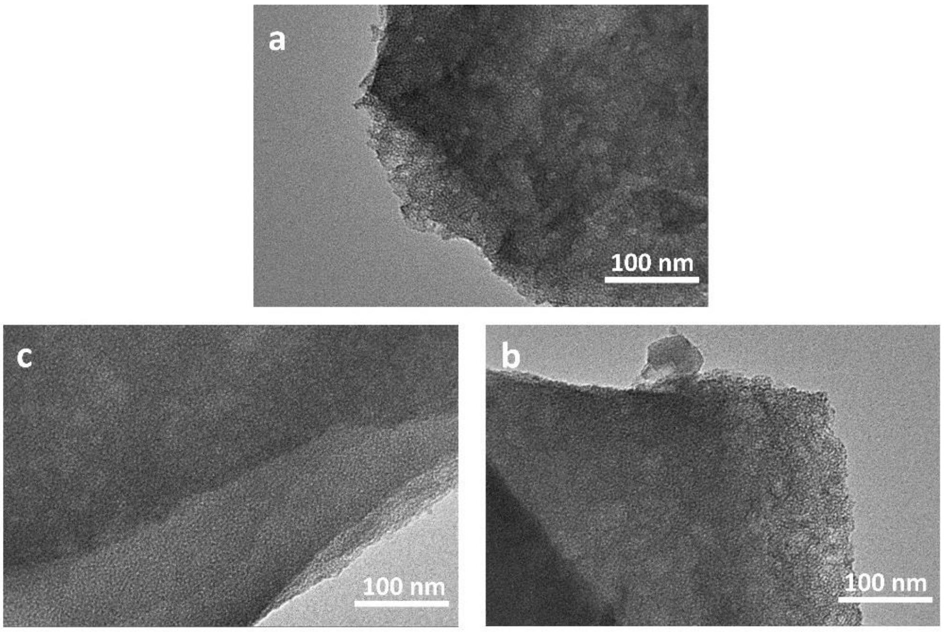

Figure S4. TEM images of catalysts. (a) Al-MCM-41(35/1), (b) AlNb-MCM-41(35/1/0.3), and (c) AlNb-MCM-41(35/1/0.7).

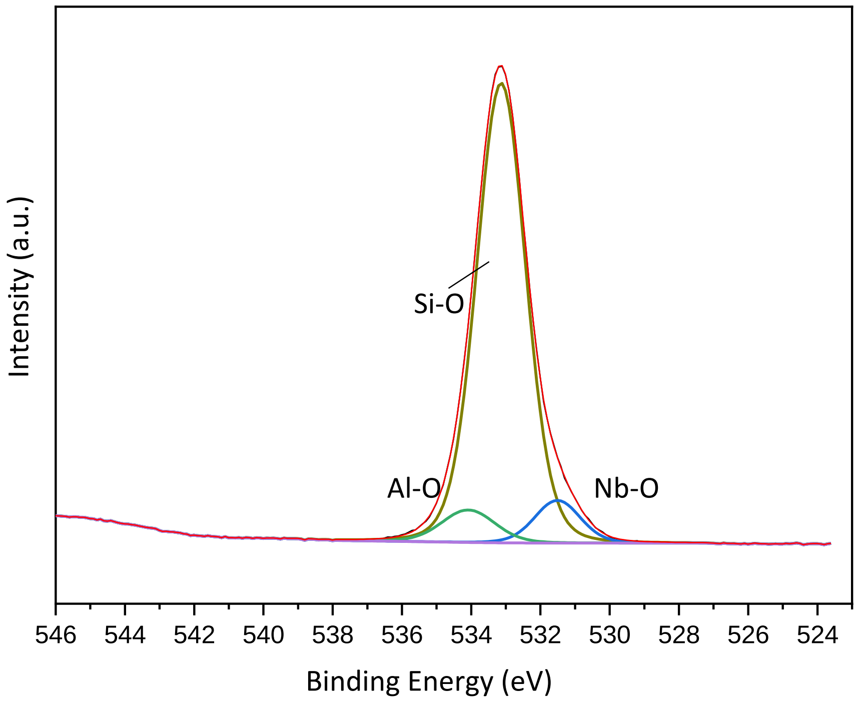

Figure S5. The O 1s deconvoluted spectrum of AlNb-MCM-41(35/1/0.9).

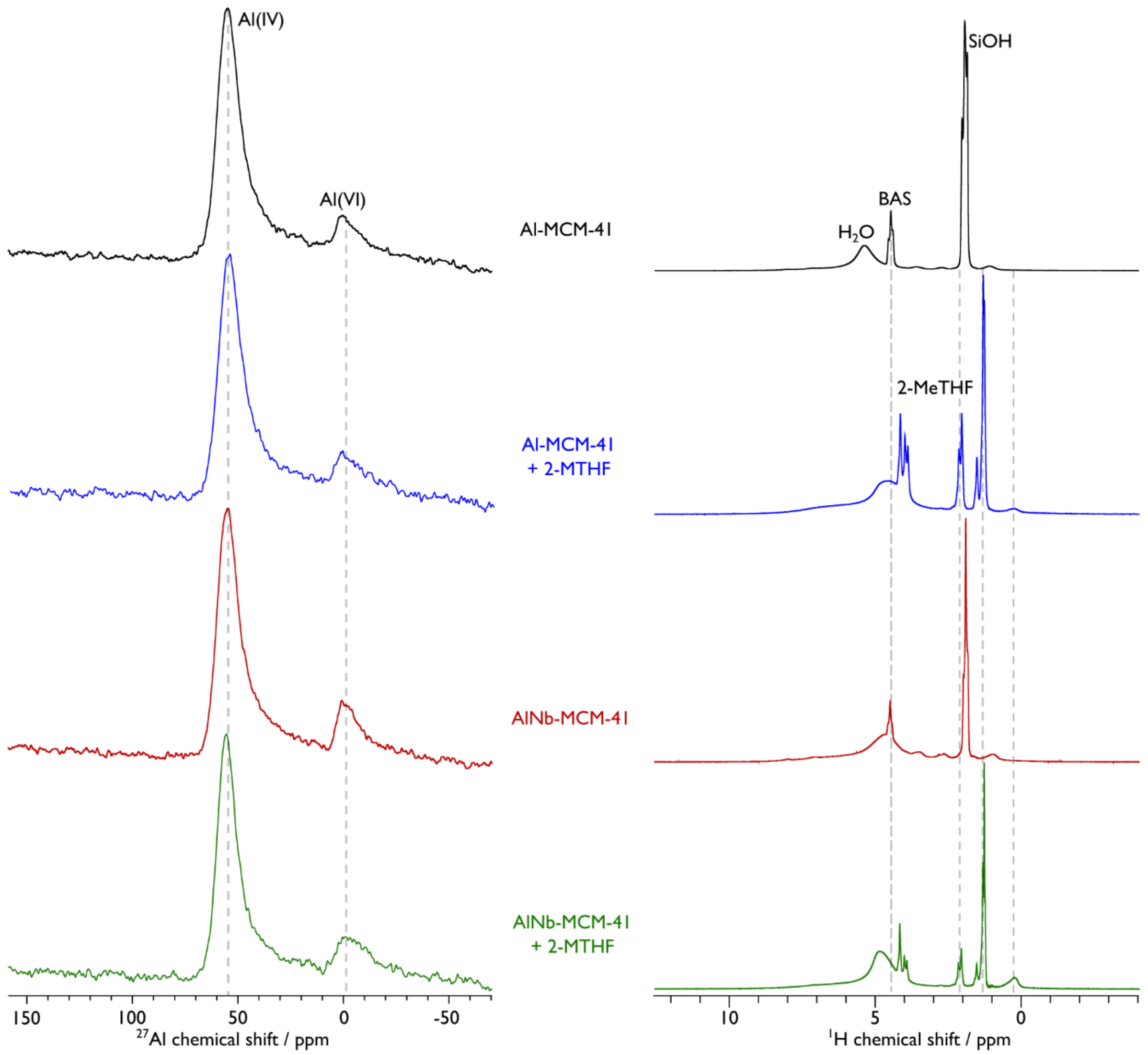

Figure S6. ^{27}Al (left) and ^{1}H (right) MAS NMR spectra of Al-MCM-41 (35/1), Al-MCM-41 (35/1) with 2-MTHF, AlNb-MCM-41(35/1/0.9) and AlNb-MCM-41(35/1/0.9) with 2-MTHF. All spectra were recorded at 20.0 T at ambient temperature and with a MAS frequency of 60 kHz.

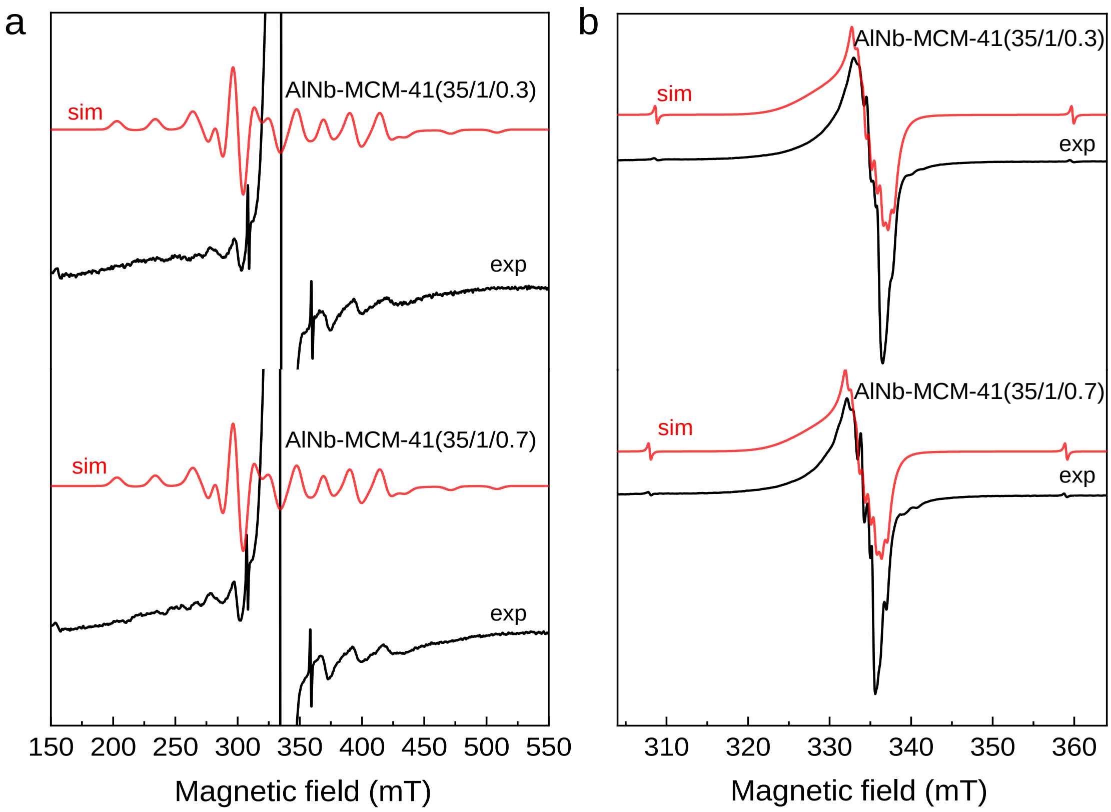

Figure S7. (a) Wide-field X-band EPR spectra of the activated molecular sieves after γ-irradiation detected at 40 K; (b) narrow-field X-band EPR spectra of the activated molecular sieves after γ-irradiation detected at 40 K. Labels are shown on the plot. Simulated spectra of Nb and Al defects are presented by the dotted line.

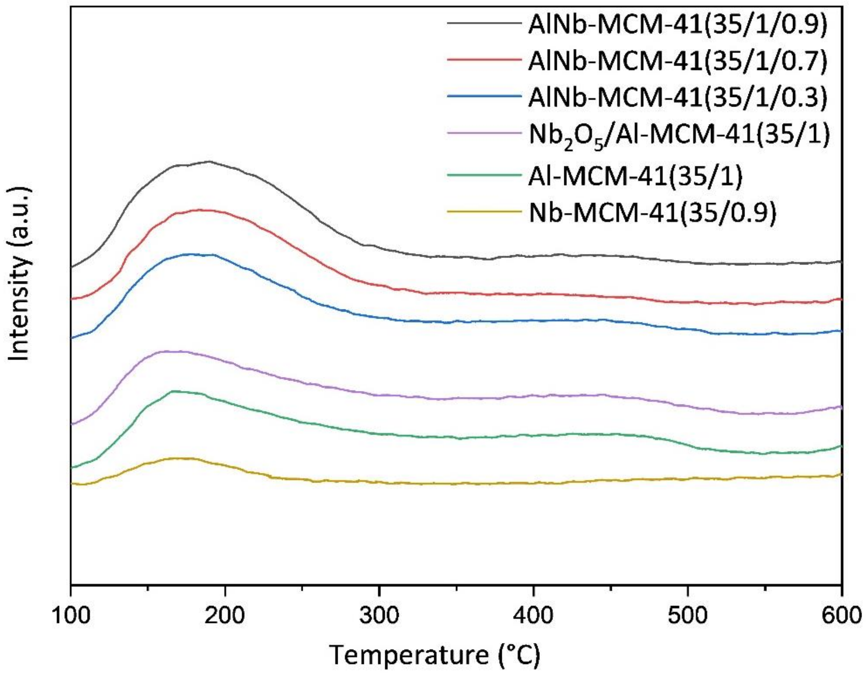

FigureS8. Characterisation of the acidity of catalysts by NH_3-TPD.

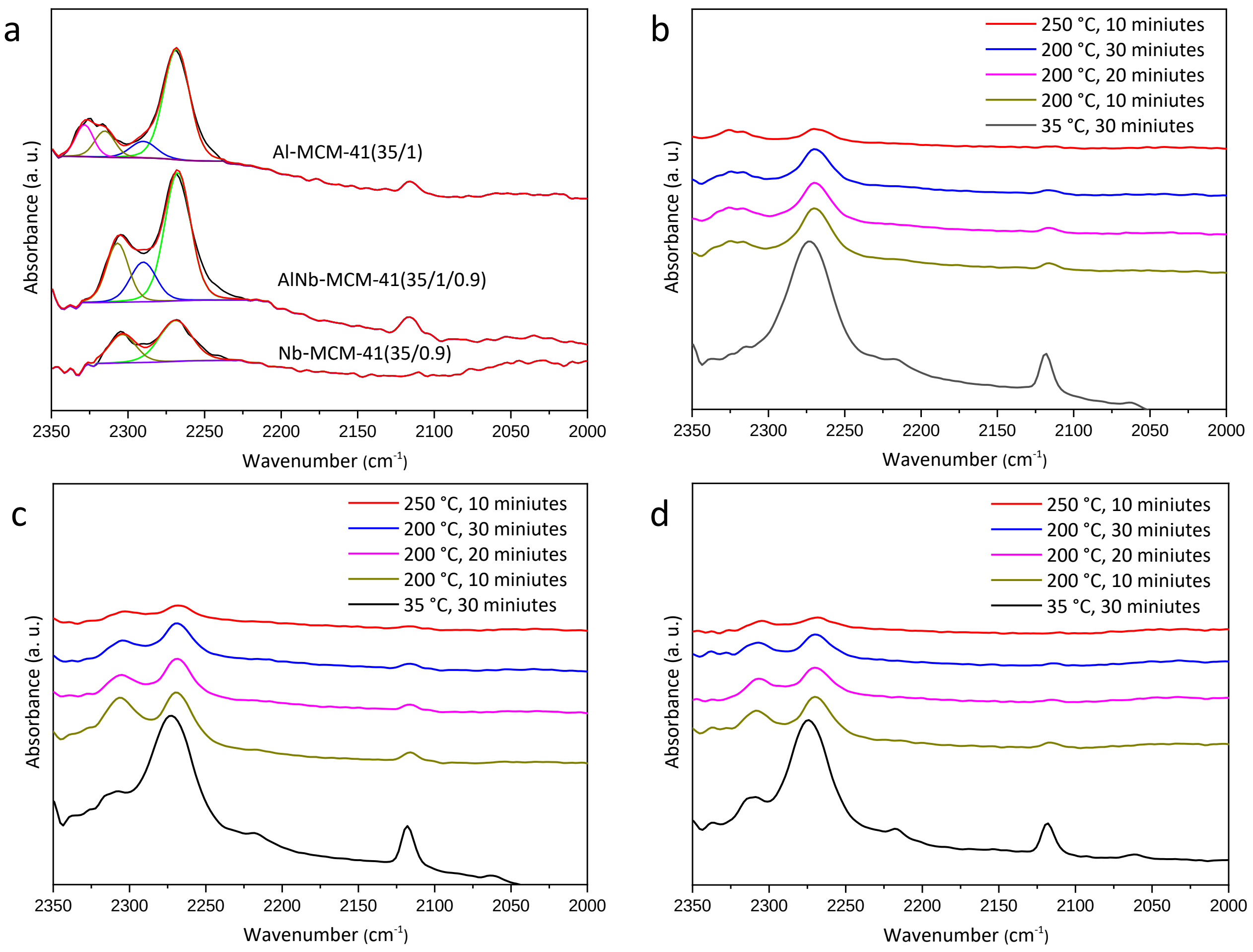

Figure S9. Characterization of the acidity of catalysts by d$_3$-acetonitrile-IR. (a) Deconvoluted d$_3$-acetonitrile-IR spectra after purge by N_2 flow for 30 minutes at 200 ^oC over Al-MCM-41(35/1), AlNb-MCM-41(35/1/0.9), and Nb-MCM-41(35/0.9). (b) Nb-MCM-41(35/0.9), (c) AlNb-MCM-41(35/1/0.9) and (d) AlNb-MCM-41 (35/1/0.9) at different temperatures and purge time. The individual components for IR peaks at 2327, 2315, 2291 and 2270 cm^{-1} are assigned to the coordinatively bound CD_3CN on strong Lewis sites, weak Lewis sites, Brønsted acid sites(bridging OH group) and terminal hydroxyls[10-12]. Deconvolution of IR spectral features was conducted by CasaXPS. Each individual peak component was centered within ±5 cm^{-1}, FWHM(full width at half maxima) were fixed to be in range of 5 and 20 cm^{-1}. The sum of Gauss-Lorentzian (SGL) line shapes were used for each component, with a 50% Lorentzian correction to a normally distributed Gaussian line shape (SGL(50)), which was used in reported work[5].

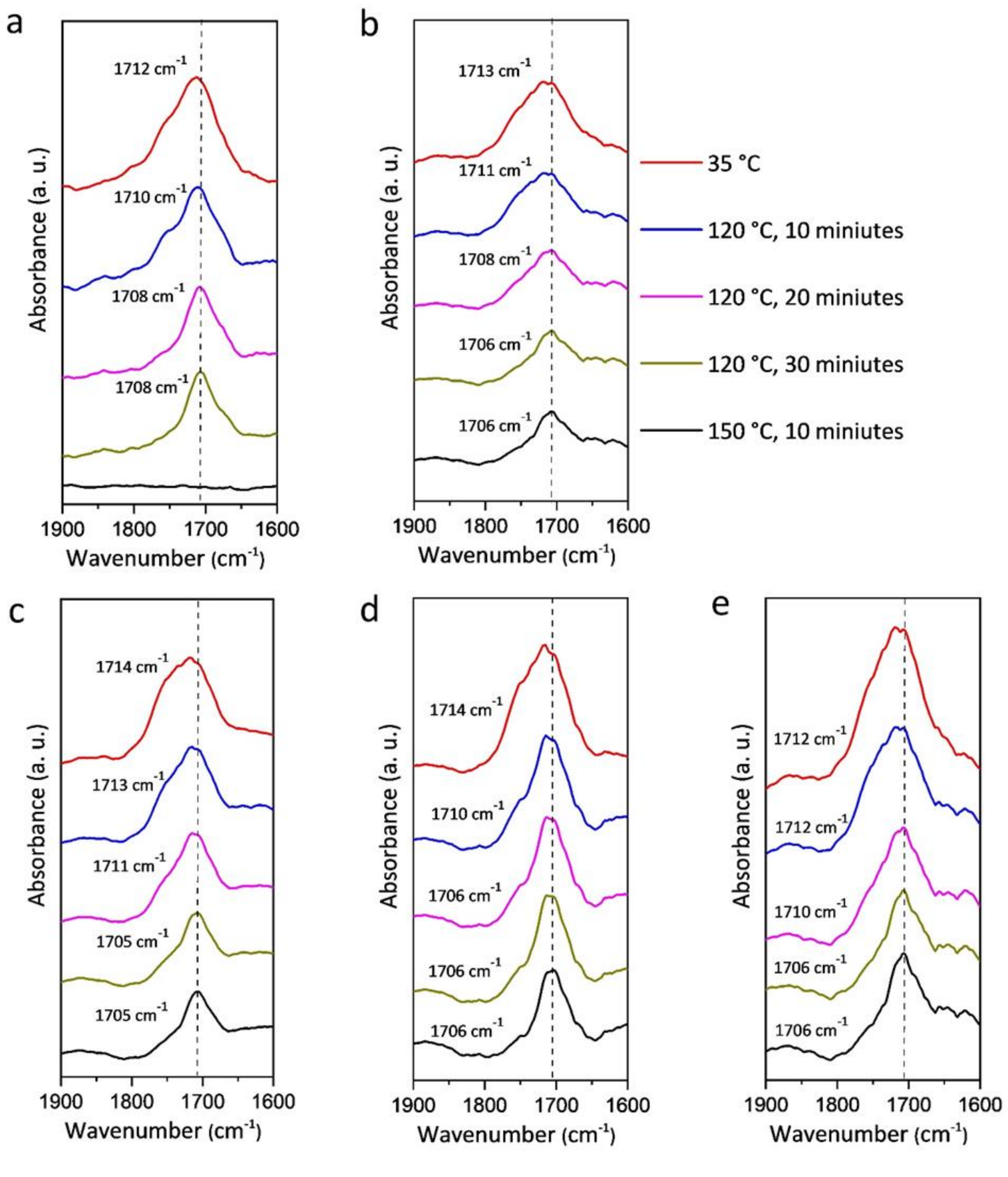

Figure S10. Acetone DRIFTS spectra. (a), Al-MCM-41(35/1), (b), Nb-MCM-41(35/0.9), (c), AlNb-MCM-41(35/1/0.9), (d), AlNb-MCM-41(35/1/0.7), (e), AlNb-MCM-41(35/1/0.3), at increasing temperatures and purge time of 35 °C, 120 °C 10 minutes, 120 °C 20 minutes, 120 °C 30 minutes, and 150 °C 10 minutes.

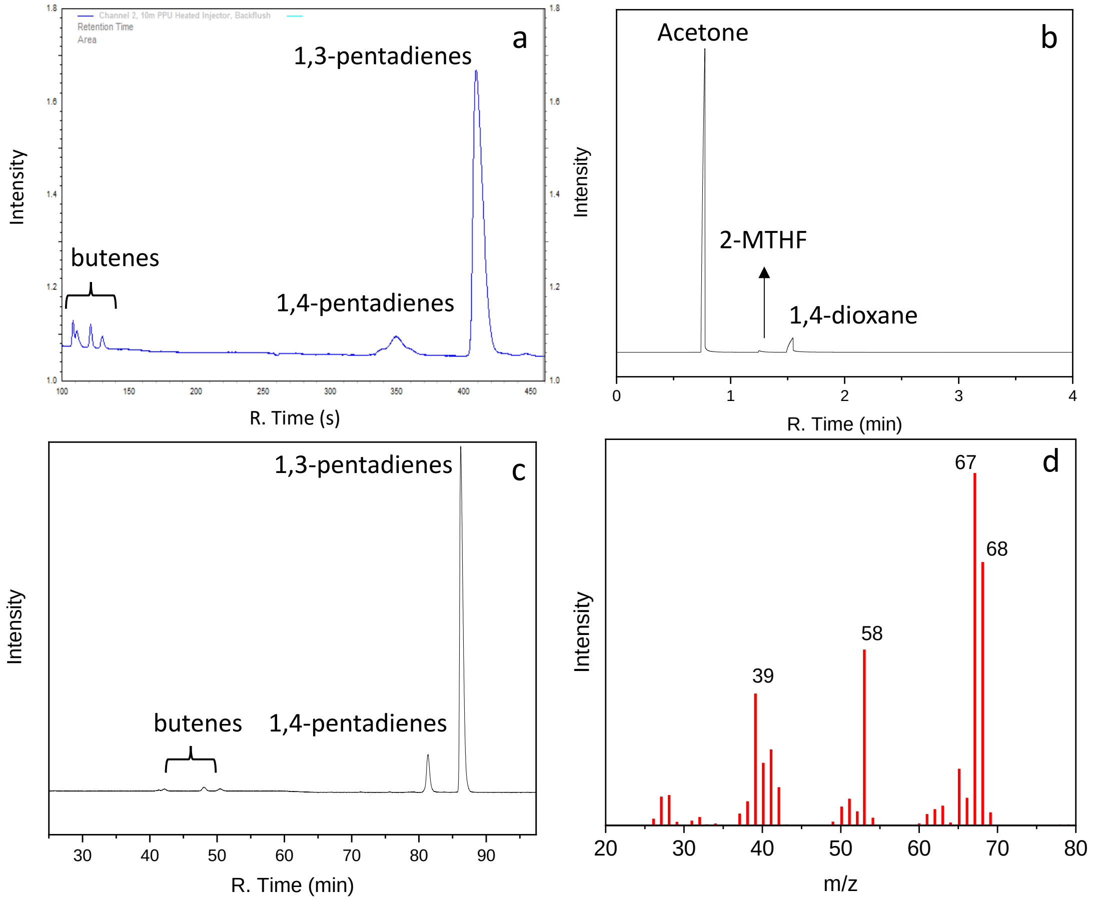

Figure S11. Analysis of the products of the conversion of 2-MTHF over 8 h on AlNb-MCM-41(35/1/0.9). (a), GC trace of the gaseous products detected by TCD, column: PLOT-U. (b), GC(FID) trace of the liquid product diluted by acetone. 1,4-Dioxane was used as the internal standard; FID, column: HP-5. It confirms that the conversion of 2-MTHF reached >99%. (c) GC-MS analysis of the gaseous products and (d) the corresponding mass spectrum of 1,3-pentadienes.

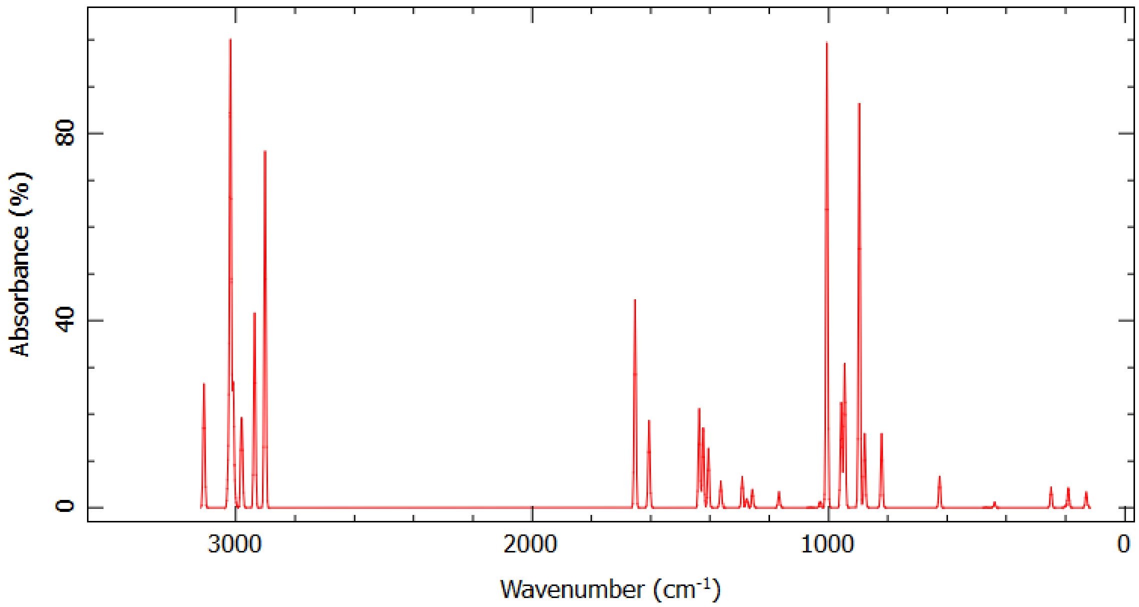

Figure S12. Simulated IR spectrum of trans-1,3-pentadiene. The simulated IR spectrum of trans-1, 3-pentadiene was obtained via DFT calculation using ORCA (version 4.2.1). Geometry optimization and consequent frequency calculation were carried out using the B3LYP functional and the def2-TZVP basis set for all atoms[13–16]. Imaginary frequency was checked and the peak frequencies corrected with a vibrational scaling factor of 0.965 for the used functional and basis set according to the Computational Chemistry Comparison and Benchmark DataBase (CCCBDB).

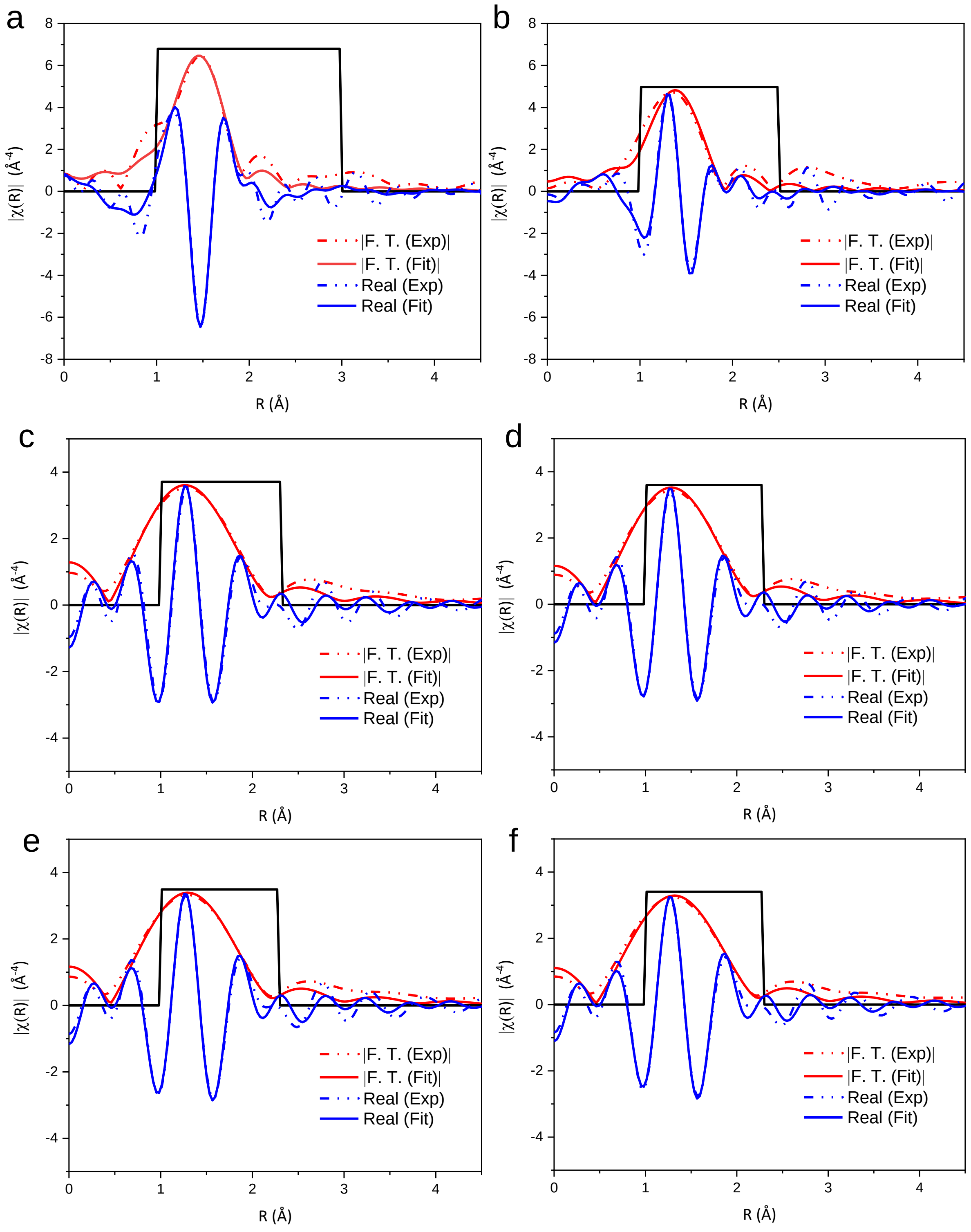

Figure S13. Fitting of EXAFS data at the Nb K-edge absorption of (a) NbAl-MCM-41(35/1/0.9), (b) 2-MTHF adsorbed AlNb-MCM-41(35/1/0.9), and reaction at (c) 125°C, (d) 175°C, (e) 225°C, (f) 275°C in R-space.

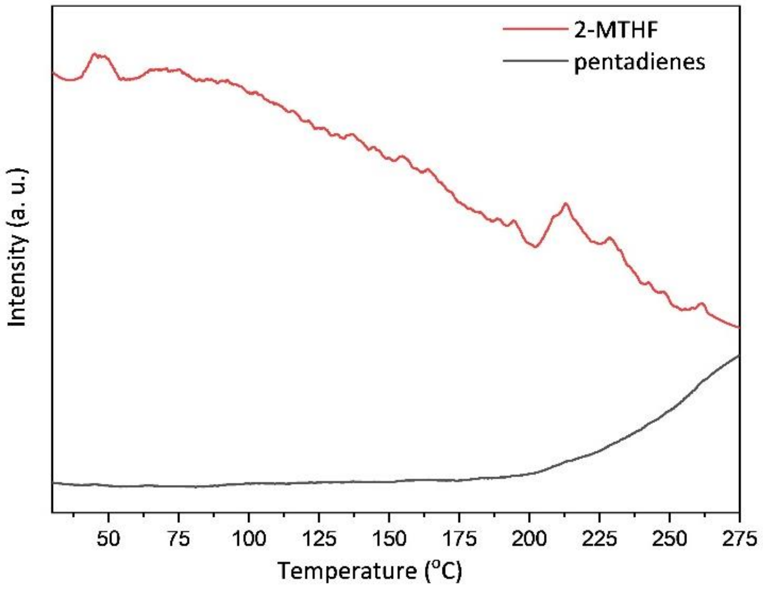

Figure S14. On-line mass spectra of 2-MTHF and pentadienes during conversion of 2-MTHF on AlNb-MCM-41(35/1/0.9), for *in situ* DRIFTS and Nb K-edge XAS experiments.

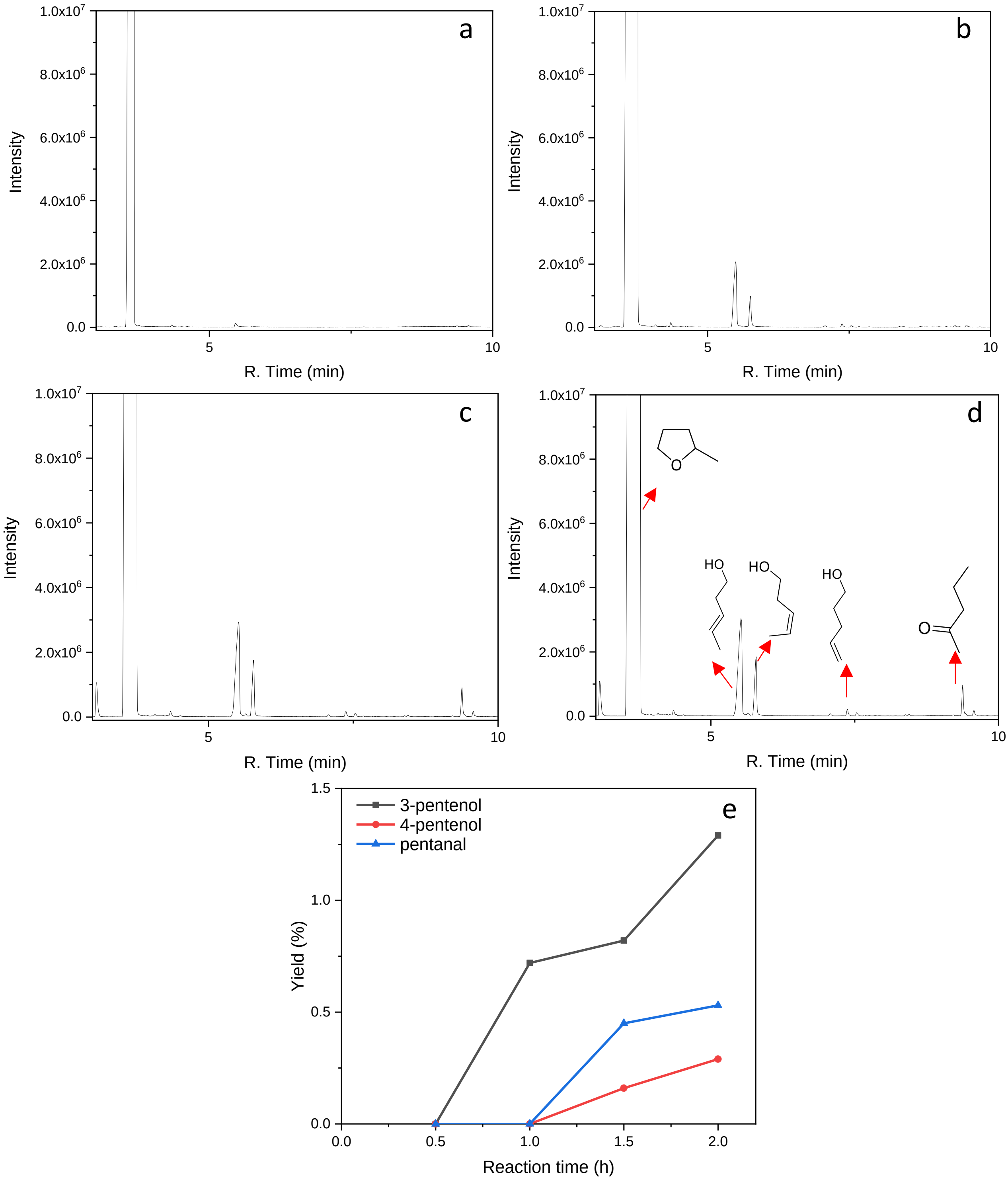

Figure S15. GC-MS analysis of the liquid products generated in the batch-mode reaction over NbAl-MCM-41(35/1/0.9) as a function of reaction time. (a) 0.5 h, (b) 1h, (c) 1.5 h, and (d) 2h. (e) Yields of different intermediates as a function of reaction time. Reaction condition: 2-MTHF, 2 ml; catalyst, 0.1 g; reaction temperature, 220 °C. Compared with the flow reaction at 275 °C, the lower reaction temperaure used in batch reactions is due the limit of the Telfon liner in the reactor.

Table S1. Specific surface areas of all zeolites used in this study (data determined from N_2 sorption isotherms at 77 K)

Samples	d_{100} (nm)[a] **[a]**	S_{BET} ($m^2 g^{-1}$)[b]	V_{total} ($cm^3 g^{-1}$)[c]	D_{BJH} (nm)[d]	t_{BJH} (nm)[e]
AlNb-MCM-41(35/1/0.3)	3.80	1209	1.00	3.01	1.38
AlNb-MCM-41(35/1/0.7)	3.75	1238	0.99	2.93	1.40
AlNb-MCM-41(35/1/0.9)	3.92	1140	1.08	3.20	1.33
Al-MCM-41(35/1)	3.82	1346	1.23	3.04	1.37

[a] Value of d_{100} of calcined samples collected from small angle PXRD patterns. [b] S_{BET} obtained based on Brunauer-Emmett-Teller (BET) equation. [c] Pore volume of the material obtained through Barrett-Joyner-Halenda (BJH) method according to the adsorption curve. d] D_{BJH} represents adsorption average pore width calculated with Barrett-Joyner-Halenda (BJH) method. [e] Pore-wall thickness calculated form equation $t_{BJH}=2d_{100}/1.732-D_{BJH}$. (Based on the relationship between unit cell a and d_{100} for 2D hexagonal space group, $d_{hkl}=[(4/3)(h^2+hk+k^2)/a^2+l^2/c^2]^{-1/2}$)

Table S2. Summary of acidity of all MCM-41 materials used in this work (data determined by NH_3-TPD)

Catalyst	Weak acid ($mmol\ g^{-1}$)	Strong acid ($mmol\ g^{-1}$)	Total acid ($mmol\ g^{-1}$)
AlNb-MCM-41(35/1/0.9)	0.258	0.024	0.282
AlNb-MCM-41(35/1/0.7)	0.231	0.024	0.255
AlNb-MCM-41(35/1/0.3)	0.186	0.024	0.21
Al-MCM-41(35/1)	0.147	0.063	0.21
Nb-MCM-41(35/0.9)	0.045	0	0.045
Nb_2O_5/Al-MCM-41(35/1)[a]	0.162	0.054	0.216

[a]The ratio of Si/Al/Nb in powdered mixture of Nb_2O_5/Al-MCM-41(35/1) (5.3wt%/94.7wt%) is 35/1/0.9.

Table S3. Spin-Hamiltonian parameters of radiation induced Nb(IV) and electron hole Al-O•-Si defects, from simulation of CW X-band EPR spectra in FigureS7: lw is the homogeneous Lorentzian linewidth, **g** and **A** are the principle values of the **g** and hyperfine interaction tensors.

Signal	Parameters					
	Electron Spin	Nuclear Spin	**g**-tensor	g-Strain	**A**-tensor (G)	lw (mT)
Nb defect	1/2	9/2	[1.936 1.936 1.877]	-	[159 159 318]	[0 10.0]
Al defect	1/2	5/2	[2.003 2.012 2.045]	[0.0 0.000 0.05]	[7 7 -]	[0.0 0.55]
1H	1/2	1/2	[2.0023 2.0023 2.0023]	-	[508 508 508]	[0.0 0.5]

Table S4. Comparison of the catalytic performance between AlNb-MCM-41 and reported catalysts for the conversion of 2-MTHF to pentadienes.

Catalyst	Reaction temperature (°C)	Conversion (%)	Selectivity (%)	TOS (h)	Reference
AlNb-MCM-41(35/1/0.9)	275	99	91	8	This work
AlNb-MCM-41(35/1/0.7)	275	99	85	8	This work
AlNb-MCM-41(35/1/0.3)	275	99	80	8	This work
Amorphous SiO_2/Al_2O_3	350	100	68	13	17
ZrO_2	300	3	100	2	18
TiO_2	300	18	89	2	18
Al_2O_3	300	22	60	2	18
HZSM-5	300	97	72	2	18
B-MFI	335	35.2	89	10	19
B-BEA	335	9.2	91	10	19
Al-BEA	335	75.7	56	10	19
B-MWW	335	74.4	89	10	19
MCM-22	335	88.1	74	10	19

.

Table S5. 1st Shell fitting of EXAFS data on the Nb sites for as-prepared and 2-MTHF-adsorbed AlNb-MCM-41.

Sample	AlNb-MCM-41	2-MTHF-adsorbed AlNb-MCM-41
Fitting range	*K* 2.6 – 10.0 $Å^{-1}$<br>R 1.0 – 3.0 Å	*K* 3.0 – 9.5 $Å^{-1}$<br>R 1.0 – 2.5 Å
Independent points	9	6
Variables	6	4
R-factor	0.019	0.017
S_0^2	1[a]	1[a]
ΔE_0(eV)	9.01±2.90	-6.55±3.16
R(Nb=O) (Å)	1.90±0.05	1.93±0.022
CN(Nb=O)	1[b]	1[b]
σ^2 (Nb=O) ($Å^2$)	0.008±0.012	0.008±0.003
R(Nb-O) (Å)	2.00±0.13	1.93±0.022
CN(Nb-O)	3.78±1.02	2.95±0.99
σ^2 (Nb-O) ($Å^2$)	0.002±0.013	0.008±0.003

[a]The amplitude factor S_0^2 was set to 1. [b]CN was set to 1 according to the structural mode

References:

1. E. Iliopoulou, E. Antonakou, S. Karakoulia, I. Vasalos, A. Lappas, K. Triantafyllidis, *Chem. Eng. J*, **2007**, *134*, 51–57.

2. Q. Xia, Q. Cuan, X. Liu, X. Gong, G. Lu, Y. Wang, *Angew. Chem. Int. Ed.* **2014**, *126*, 9913–9918.

3. S. Stoll, A. Schweiger, *J. Magn. Reson.* **2006**, *178*, 42–55.

4. D. Massiot, F. Fayon, M. Capron, I. King, S. Le Calvé, B. Alonso, J-O. Durand, B. Bujoli, Z. Gan, G. Hoatson, *Magnetic resonance in chemistry*. **2002**, *40*, 70-76.

5.a) S. Diaz-Moreno, M. Amboage, M. Basham, R. Boada, N. E. Bricknell, G. Cibin, T, M. Cobb, J. Filik, A. Freeman, K. Geraki, D. Gianolio, S. Hayama, K. Ignatyev, L. Keenan, I. Mikulska, J. F. W. Mosselmans, J. J. Mudd, S. A. Parry, *J. Synchrotron Radiat*. **2018**, *25*, 998-1009. b) S. Diaz-Moreno, S. Hayama, M. Amboage, A. Freeman, A. Sutter, G. Duller, *J. Phys.: Conf. Ser.* **2009**, *190*, 012038.

6. H. Tolentino, E. Dartyge, A. Fontaine, G. Tourillon, *J. Appl. Cryst.* **1988,** *21*. 15-22.

7. J. C. Labiche, O. Mathon, S. Pascarelli, M. A. Newton, G. G. Ferre, C. Curfs, G. Vaughan, A. Homs, D. F. Carreiras, *Rev. Sci. Instrum*. **2007**, *78*, 091301.

8. M. Basham, J. Filik, M. T. Wharmby, P. C. Y. Chang, B. E. Kassaby, M. Gerring, J. Aishima, K. Levik, B. C. A. Pulford, I, Sikharulidze, *J. Synchrotron Rad.* **2015**, *22*, 853-858.

9. B. Ravel, M. Newville, *J. Synchrotron Rad.* **2005**, *12*, 537–541.

10. O. Bortnovsky, Z. Sobalík, B. Wichterlová, Z. Bastl, *J. Catal.*, **2002**, *210*, 171–182.

11. A. Pelmenschikov, R. van Santen, J. Janchen, E. Meijer, *J. Phys. Chem.* **1993**, *97*, 11071–11074.

12. J. Harris, M. Cordon, J. Di Iorio, J. Vega-Vila, F. Ribeiro, *J. Catal.*, **2016**, *335*, 141–154.

13. F. Neese, *WIREs Comput. Mol. Sci.*, **2012**, *2*, 73–78.

14. F. Neese, *WIREs Comput. Mol. Sci.*, **2018**, *8*, e1327.

15. F. Weigend, R. Ahlrichs, *Phys. Chem. Chem. Phys.*, **2005**, *7*, 3297–3305.

16. G. Stoychev, A. Auer, F. Neese, *J. Chem. Theory Comput.* **2017**, *13*, 554–562.

17. M. Kumbhalkar, J. Buchanan, G. Huber, J. Dumesic, *ACS Catal.* **2017**, *7*, 5248–5256.

18. Y. Ji, S. Batchu, A. Lawal, D. Vlachos, R. Gorte, S. Caratzoulas, O. Abdelrahman, *J. Catal.* **2022**, *410*, 10–21.

19. G. Kumar, D. Liu, D. Xu, L. Ren, M. Tsapatsis, P. J. Dauenhauer, *Green Chem.* **2020**, *22*, 4147–4160.
